# Assessment of mealtime behaviors in Chinese children with autism spectrum disorder

**DOI:** 10.3389/fped.2025.1597023

**Published:** 2025-06-12

**Authors:** Lili Zhang, Yunjian Zhang, Zhongbi Peng, Yun Chen, Lan Zhang, Lidan Liang, Yi Wang, Hao Zhou

**Affiliations:** ^1^ Department of Pediatrics, Minhang Hospital, Fudan University, Shanghai, China; ^2^ Department of Neurology, National Children’s Medical Center, Children’s Hospital of Fudan University, Shanghai, China; ^3^ Department of Neurological Rehabilitation, Guizhou Provincial People’s Hospital, Medical College of Guizhou University, Guiyang, China; ^4^ Child Health Care, Chengdu Women’s and Children’s Central Hospital, Chengdu, China; ^5^ Department of Children's Rehabilitation, The Second Affiliated Hospital and Yuying Children’s Hospital of Wenzhou Medical University, Wenzhou, China; ^6^ Department of Rehabilitation, National Children’s Medical Center, Children’s Hospital of Fudan University, Shanghai, China

**Keywords:** autism spectrum disorder, reliability and validity, dietary behavior, brief autism mealtime behavior inventory, gastrointestinal problem

## Abstract

**Background:**

Children with autism often exhibit atypical eating behaviors, which may significantly impact their nutritional status and overall well-being. However, research on the dietary habits of Chinese children with autism remains limited.

**Objective:**

This study primarily assesses the applicability of Brief Autism Mealtime Behavior Inventory (BAMBI) for Chinese children with autism, while further exploring the unique dietary behavior characteristics within this population.

**Methods:**

In total, 103 children with autism aged 3–6 years and 331 typically developing preschoolers were recruited. All participants’ parents completed the Brief Autism Mealtime Behavior Inventory (BAMBI) and the Six-item Gastrointestinal Severity Index (6-GSI). The reliability and validity of the Chinese version of the BAMBI were analyzed, and differences in dietary behavior were tested between cases and controls.

**Results:**

The item reliability (Cronbach's alpha) was 0.849 for total BAMBI. Pearson correlation analyses of the full BAMBI scale scores and subscales revealed significant correlations (r values ranging from 0.580 to 0.912, *P* < 0.01). The confirmatory factor analysis result shows that BAMBI 18 items have an acceptable fit to the data (GFI = 0.901, CFI = 0.819, ILI = 0.821, RMSEA = 0.074). Although the value of average variance extra (AVE) of the main scale is slightly below 0.5, the composite reliability (CR) is both above 0.7. The ASD group scored significantly higher than the typically developing group in terms of the BAMBI total score and subscales. The top problematic dietary behaviors reported by parents were inflexible about mealtime routines (79.61%), unwilling to try new foods (75.73%) and not accepts or prefers a variety of foods (71.84%). The positive association between BAMBI total scores and 6-GSI scores was observed.

**Conclusion:**

BAMBI is a validated tool for assessing dietary behaviors in autistic children in China. Compared with their typically developing peers, ASD children presented more dietary behavior problems. This study emphasizes early attention to dietary behavior problems in children with autism.

## 1 Introduction

Autism spectrum disorder (ASD) is a neurodevelopmental disorder characterized by impaired social interactions, communication and repetitive behaviors (1). The prevalence of ASD has been increasing globally with about 787% increase over 20 years (2–4) and now the reported global prevalence is 100/10,000 (2). This trend is no different in mainland China. The latest estimated prevalence of ASD in China is 0.70% (5), making it a significant public health concern. The increase in prevalence has led to greater awareness, and many studies have focused on exploring the various aspects of autism, including dietary behaviors (6–9).

Dietary behaviors in children with autism have gained much more attention for several reasons. First, more than half of children with ASD exhibit selective eating patterns (10, 11), food aversions (12), and sensory sensitivities (13) that can lead to nutritional imbalances (14). These behaviors can have a profound impact on their overall health, growth, and development. Second, a growing body of evidence suggests that dietary interventions may play a role in managing some of the symptoms associated with autism, such as gastrointestinal issues (15) and behavioral challenges (16). However, most of the existing studies have been conducted in Western countries, and data on the specific dietary behaviors of Chinese children with ASD are lacking.

Researchers have developed questionnaires to evaluate dietary routines, attitudes, and food practices, as quick and clinically useful assessments. Currently available parent-report questionnaires for assessing dietary behaviors in children with ASD include the Behavioral Pediatrics Feeding Assessment Scale (17) (originally designed for children with cystic fibrosis), the Children's Eating Behavior Inventory (18) (created for various medical populations), and Meals in Our Household (19) (originally designed for children with developmental delays). However, none of these questionnaires are specific to ASD children. The Brief Autism Mealtime Behavior Inventory (BAMBI) was developed as the first standardized informant report measure to specifically assess dietary behaviors in children with ASD (20) with adequate psychometric properties (21) and statistics (22, 23) reported.

Therefore, in this study, we introduced the BAMBI to assess the dietary behaviors of children with autism to identify specific eating habits in the Chinese context, and the reliability and validity of the Chinese version of the BAMBI were analyzed before its use in these populations.

## 2 Methods

### 2.1 Ethical approval

The present investigation was approved by the Ethics Board of Guizhou Provincial People's Hospital (2024-138). Oral informed consent was obtained from all parents. Caregivers of all eligible subjects were then invited with consent to participate in this study.

### 2.2 Participants

#### 2.2.1 Sample size estimation

According to rough estimate of sample size of Kendall (24), the sample size for the questionnaire should be 5 to 10 times the number of items on the scale. Since BAMBI has a total of 18 items, the estimated required sample size ranges from 90 to 180 samples. Considering a 10% rate of invalid questionnaires and the stability of statistical analysis, the final sample size should be greater than 100 participants.

#### 2.2.2 Children with ASD

This study was conducted from June 2024 to August 2024. The ASD population was obtained from four sites, including the Rehabilitation Department of Pediatrics at the Children's Hospital of Fudan University, the Rehabilitation Department of Pediatrics at Guizhou Provincial People's Hospital, The Second Affiliated Hospital and Yuying Children's Hospital of Wenzhou Medical University and Child Health Care of Chengdu Women and Children's Hospital. All the ASD patients: (1) meeting the criteria of the Diagnostic and Statistical Manual of Mental Disorders, Fifth Edition (DSM-5); (2) the age was from 3 to 6 years old; (3) parents of children with autism agreed to participate in this study.

#### 2.2.3 Control group

A convenience sample of children with typical development was recruited from the public kindergarten. The inclusion criteria of the typically developing group were age matched with the ASD group, but without any mental, neurological, or metabolic disease based on the child health record provided by the kindergarten.

### 2.3 Measures

#### 2.3.1 The brief autism mealtime behavior inventory

The Brief Autism Mealtime Behavior Inventory (BAMBI) is a specialized tool developed to assess mealtime behaviors in children with ASD aged 3–11 years. It was first introduced in 2008 and has since been validated and adapted for use in various populations, including Turkish (25), Brazilian Portuguese (21), and other (26) populations.

The BAMBI questionnaire includes 18 items and is divided into 3 subscales (Limited Variety, Food Refusal and Features of Autism). The frequencies were scored on a 1–5 Likert scale, with a score of 1 indicating that the behavior “never” occurred and a score of 5 indicating that the behavior “always” occurred at mealtime, to quantify the autistic traits. Four of the items were reverse scored, as they were used to rate positive mealtime behaviors. The BAMBI total score was calculated from a sum of all 18 items, with higher scores reflecting more mealtime behavior problems. The subscale score was calculated by summing the scores of the included items.

Our team adopted standard translation and back-translation procedures to translate the Chinese version of the BAMBI. Before the study began, we recruited a few parents of ASD patients aged 3–6 years from the outpatient clinic of the Children's Hospital of Fudan University to complete the Chinese version of the BAMBI, as this would ensure that the questions and concepts were clear and could accurately capture the mealtime behaviors of children with ASD in the Chinese context. The fact that the participants found the context of the Chinese version of the BAMBI understandable suggests that the translation and cultural adaptation processes were successful; this made it possible to further study the Chinese version BAMBI.

#### 2.3.2 The six-item gastrointestinal severity index

The Six-item Gastrointestinal Severity Index (6-GSI) was developed by Schneider in 2006 and is used to assess 6 GI symptoms, including constipation, stool odor, diarrhea, stool consistency, flatulence and abdominal pain (27). Each symptom was assigned a score of 0, 1, or 2 on the basis of its frequency per week. A score of 0 was interpreted as the absence of symptoms, whereas scores of 1 and 2 denoted the presence of symptoms of differing severity. The GI score was the sum of the six items, with higher scores indicating more serious GI symptoms.

#### 2.3.3 Data collection

We used online surveys as methodology to collect demographic and questionnaire data via the WeChat network for both children with typical development and children with ASD. Demographic data including age, sex, ethnic group, height, and weight, residence, family income, parental education level, and parental occupation were collected by self-administered questionnaire.

### 2.4 Statistical analysis

Data analysis was performed via IBM SPSS version 27.0 (IBM SPSS, Inc., Chicago, IL, USA) and AMOS 22. Cronbach's alpha was employed to test the internal consistency of each subscale (28). For the BAMBI, each item with a score of ≥3 was considered to indicate problematic behavior. Descriptive statistics were performed in total and by group. Characteristics of study participants, including all scores were expressed as the mean (SD) for normally distributed variables, median (IQR) for skewed variables and frequency with percentage for categorical variables. Group comparisons were performed by 2-sample *t*-test, Kruskal–Wallis test and chi-square test, respectively.

Due to the low Cronbach's alpha coefficient of the Feature of Autism, which indicates that it is not sufficiently reliable, we did not conduct further component analysis on it.

Linear regression models were performed to evaluate the association between mealtime behavior and GI symptoms.

All tests were two-tailed, and a *P* value of less than 0.05 was considered statistically significant.

## 3 Results

### 3.1 Demographic characteristics of the current samples

In total, 108 (94.74%) BAMBI questionnaires from the four sites and 352 (87.56%) from the kindergarten were collected. 5 questionnaires from ASD cases and 21 from typically developing children were not included in the final analysis because of missing items or basic information (especially the date of birth). We obtained a final sample of 434 children aged 3‒6 years, including 103 children with ASD (mean age 5.18 ± 1.40 years, 87 males) and 331 age-matched controls (mean age 5.46 ± 0.76 years, 171 males). No statistically significant differences were observed in age, BMI, ethnic group, residence, family income, or mother's occupation between the two groups. Significant differences in gender, father's occupation and parental education were observed between the two groups (*P* < 0.01) (Table 1).

**Table 1:** Demographic characteristics of participants in the TD and ASD groups.

Characteristics	Category	TD (*N* = 331)	ASD (*N* = 103)	*χ*^2^/*t*	*P* value^a^
		*N* (%)	*N* (%)		
Age (year)^b^	Mean	5.46 ± 0.76	5.18 ± 1.40	1.905	0.059
BMI^b^	Mean	17.44 ± 5.00	17.76 ± 4.50	0.569	0.569
Gender	Male	171 (51.7)	87 (84.5)	35.066	<0.001
	Female	160 (48.3)	16 (15.5)		
Ethnic groups	Ethnic Han	237 (71.6)	66 (64.1)	2.110	0.146
	Ethnic minorities	94 (28.4)	37 (35.9)		
Residence	Urban area	117 (35.5)	42 (40.8)	0.997	0.318
	Rural area	214 (64.7)	61 (59.2)		
Family Income (monthly)	Below 5,000 RMB	153 (46.2)	46 (44.7)	0.13	0.937
	5,000–10,000 RMB	143 (43.2)	45 (43.7)		
	Above 10,000 RMB	35 (10.6)	12 (11.7)		
Father's occupation	Civil servant	9 (2.7)	8 (7.8)	12.189	0.007
	Tech worker	63 (19.0)	28 (27.2)		
	Freelancer	72 (21.8)	26 (25.2)		
	other	187 (56.5)	41 (39.8)		
Mother's occupation	Civil servant	6 (1.8)	6 (5.8)	5.925	0.115
	Tech worker	46 (13.9)	17 (16.5)		
	Freelancer	85 (25.7)	28 (27.2)		
	other	194 (58.6)	52 (50.5)		
Father's education	Undergraduate	263 (79.5)	69 (67.0)	12.849	0.002
	Bachelors	65 (19.6)	28 (27.2)		
	Postgraduate	3 (0.9)	6 (5.8)		
Mother's education	Undergraduate	266 (80.4)	72 (69.9)	12.832	0.002
	Bachelors	65 (19.6)	28 (27.2)		
	Postgraduate	0 (0)	3 (2.9)		

^a^ Performed by 2-sample *t*-test for normally distributed variables and chi-square test for categorical variables.

^b^ Values are mean (±SD).

TD, typically developing; ASD, autism spectrum disorder.

### 3.2 Reliability and validity of the Chinese version of the BAMBI

**Figure 1:**
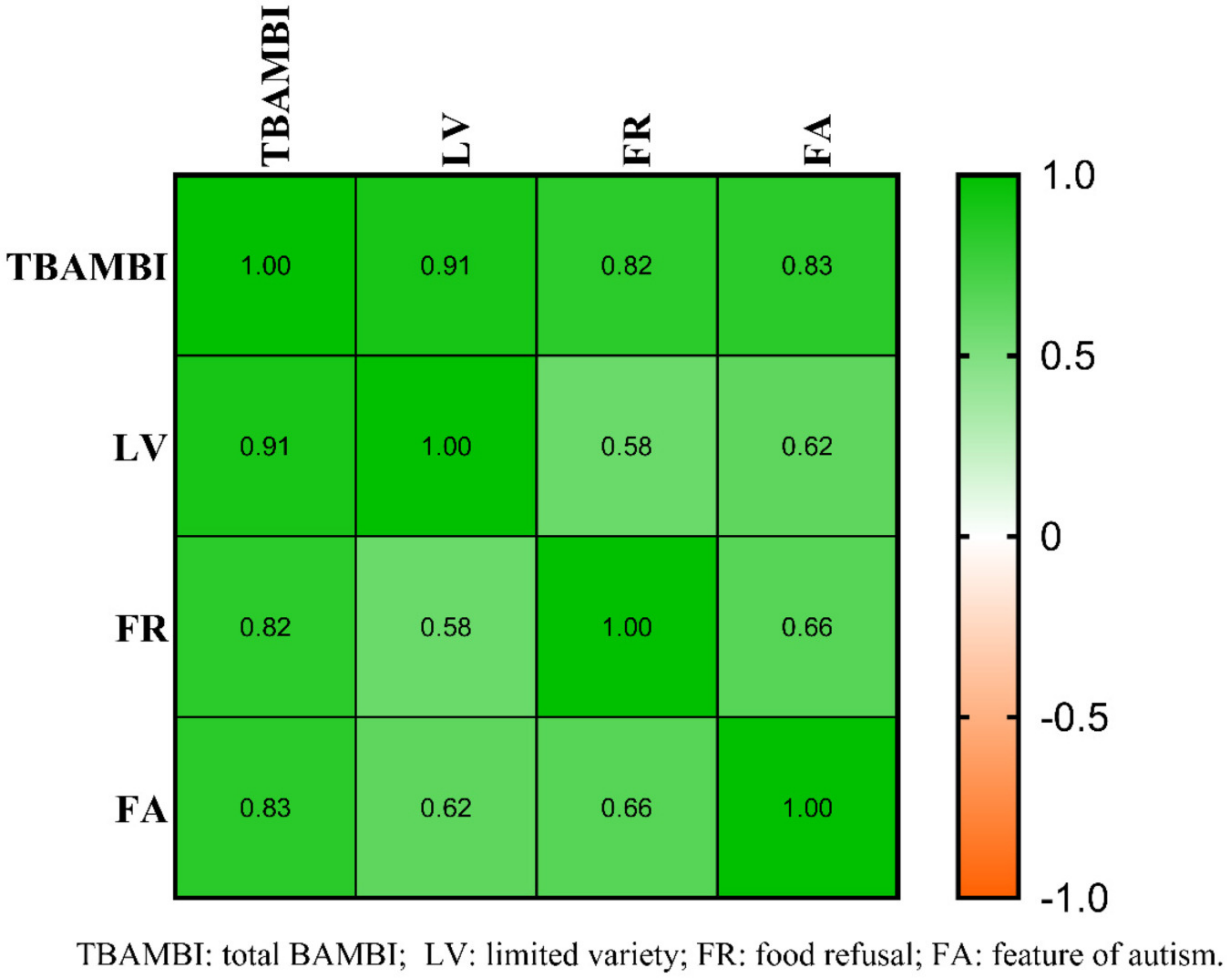
Correlation matrix of BAMBI.

The scale's reliability is assessed through internal consistency by using Cronbach's alpha. Cronbach's alpha of the total BAMBI was 0.849, indicating good internal consistency for the full scale (Table 2). Pearson correlation analyses of the total BAMBI scale and subscales revealed significant correlations with r values ranging from 0.580 to 0.912 (*P* < 0.01, Figure 1).

**Table 2:** Cronbach's *α* and confirmatory factor analysis in Chinese version BAMBI.

BAMBI	Cronbach's *α* (*N* = 434)	GFI	CFI	IFI	RMSEA	CR	AVE
Total BAMBI	0.849	0.829	0.819	0.821	0.074	–	–
Limited Variety	0.744	–	–	–	–	0.747	0.379
Food Refusal	0.734	–	–	–	–	0.736	0.361
Feature of Autism	0.455	–	–	–	–	–	–

BAMBI, brief autism mealtime behavior inventory; GFI, goodness of fit index; CFI, comparative fit index; IFI, incremental fit index; RMSEA, root mean square error of approximation; CR, composite reliability; AVE, average variance extracted.

The confirmatory factor analysis demonstrated that the values for fit indices fell within the acceptable to good ranges (GFI = 0.901, CFI = 0.819, IFI = 0.821, RMSEA = 0.074). The composite reliability (CR) values and average variance extracted (AVE) for the two dimensions of LV and FR are 0.747, 0.736 and 0.379, 0.361, respectively (Table 2).

Scores between typically developing children and ASD cases were compared to test the discriminant ability of BAMBI. The ASD group scored significantly higher than the typically developing group in total BAMBI and subscales (Table 3).

**Table 3:** Scores between TD group and ASD group^a^.

BAMBI	TD (*N* = 331)	ASD (*N* = 103)	*H*	*P* value^b^
Total BAMBI	32 (27, 40)	41 (35, 47)	57.88	<0.001
Limited Variety	17 (14, 21)	22 (18, 25)	36.74	0.001
Food Refusal	7 (6, 9)	8 (7, 12)	16.94	<0.001
Feature of Autism	8 (6, 9)	11 (9, 13)	103.33	<0.001

^a^ Values are median (IQR).

^b^ Equality between groups by Kruskal–Wallis equality-of-populations rank test.

BAMBI, brief autism mealtime behavior inventory; TD, typically developing; ASD, autism spectrum disorder.

### 3.3 Comparison of problematic mealtime behaviors between groups

Referring to 34 as the BAMBI cutoff point (29), 76.7% of problematic feeders were identified in ASD group. Compared with typically developing children, children with autism were significantly more likely to exhibit 10 problematic behaviors (Table 4).

**Table 4:** Problematic mealtime behaviors between TD group and ASD group.

Mealtime behaviors	TD (%)	ASD (%)	χ^2^	*P* value^a^
Q1: Cries or screams during mealtimes	35 (10.57)	25 (24.27)	12.37	<0.001
Q2: Turns his/her face or body away from food	54 (16.31)	35 (33.98)	15.04	<0.001
Q3: Not remain seated at the table until the meal is finished	87 (26.28)	51 (49.51)	19.55	<0.001
Q4: Expels (spits out) food that he/she has eaten	43 (12.99)	22 (21.36)	4.32	0.038
Q5: Is aggressive during mealtimes (hitting, kicking, scratching others)	14 (4.23)	4 (3.88)	0.02	0.878
Q6: Displays self-injurious behavior during mealtimes (hitting self, biting self)	0	1 (0.97)	-	0.013
Q7: Is disruptive during mealtimes (pushing/throwing utensils, food)	51 (15.41)	21 (20.39)	1.41	0.235
Q8: Closes his/her mouth tightly when food is presented	33 (9.97)	17 (16.50)	3.29	0.070
Q9: Inflexible about mealtime routines (e.g., times for meals, seating arrangements, place settings)	101 (30.51)	82 (79.61)	77.65	<0.001
Q10: Not willing to try new foods	93 (28.10)	78 (75.73)	74.64	<0.001
Q11: Dislikes certain foods and won’t eat them	103 (31.12)	37 (35.92)	0.83	0.362
Q12: Refuses to eat foods that require a lot of chewing (e.g., eats only soft or pureed foods)	48 (14.50)	33 (32.04)	15.92	<0.001
Q13: Prefers the same foods at each meal	98 (29.61)	43 (41.75)	5.28	0.022
Q14: Prefers “crunchy” foods (e.g., snacks, crackers)	99 (29.91)	50 (48.54)	12.10	0.001
Q15: Not accepts or prefers a variety of foods	81 (24.47)	74 (71.84)	76.79	<0.001
Q16: Prefers to have food served in a particular way	92 (27.79)	33 (32.04)	0.69	0.406
Q17: Prefers only sweet foods (e.g., candy, sugary cereals)	185 (55.89)	63 (61.17)	0.89	0.345
Q18: Prefers food prepared in a particular way (e.g., eats mostly fried foods, cold cereals, raw vegetables)	118 (35.65)	44 (42.72)	1.68	0.195

^a^ Performed by chi-square test or Fisher's Exact Test.

TD, typically developing; ASD, autism spectrum disorder.

The top 3 problematic mealtime behaviors were as follows: inflexible about mealtime routines (79.61%), unwilling to try new foods (75.73%) and not accepts or prefers a variety of foods (71.84%).

### 3.4 Association between mealtime behaviors and GI symptoms

The multivariable regression model revealed positive association between BAMBI total score and 6-GSI scores (*β* = 3.95, z = 12.38, 95% CI: 3.32–4.58, *P* < 0.001) after adjusting age, gender, parental education levels (postgraduate, bachelor, undergraduate), and parental occupations (civil servant, tech worker, freelancer, others).

## 4 Discussion

In this study, a general reliability and validity test on BAMBI was performed to ensure its applicability in a Chinese context. The results of the reliability and validity tests suggest that it can be used as a dietary behavior screening tool for children with autism in China. Through BAMBI, we identified 76.7% of ASD presenting dietary behavior issues in China, which is consistent with previous research (29). These behaviors correspond to varying degrees of gastrointestinal symptoms. Therefore, early use of targeted assessment tools to evaluate dietary behaviors in children with autism is an important aspect of a comprehensive assessment of autism.

Our study revealed Cronbach's coefficient of 0.849 for total BAMBI, indicating good internal consistency of the BAMBI. The 2 primary outcome subscales also indicated good reliability: 0.744 for Limited Variety and 0.734 for Food Refusal. Because of the lower coefficient alpha of 0.455 for Feature of Autism (26), it has been excluded from further analyses. Although the confirmatory factor analysis results were not reported in other articles on BAMBI (26), we conducted a structural validity analysis for the Chinese version BAMBI. The correlation analysis demonstrated better r values (r: 580–0.912) than did the original data (r: 0.45–0.54) (20). While the value of average variance extracted (AVE) and composite reliability (CR) for the 2 main scales were found to be more than 0.36 and 0.70 respectively, indicating the presence of convergent and discriminant validity for BAMBI. However, further research through larger sample for BAMBI is still needed to verify its usage in Chinese culture.

Consistent with previous research, our data also showed severe food selectivity behaviors in children with autism in China. Our findings revealed that 2 out of the top 3 items belong to the Limited Variety, indicating a strong preference for certain foods among affected children. Although some reports suggest that food selectivity in children with ASD is associated with sensory sensitivity (30, 31), more research indicates a stronger correlation with stereotyped behaviors in these children. The effectiveness of behavioral intervention, such as the food chaining method (32), in treating food selectivity in children has been confirmed (33, 34), highlighting the potential clinical value of behavior management in addressing dietary issues. Besides, the higher incidence of problematic mealtime behaviors in Chinese ASD children was also observed compared to the Chinese American children in the United States (35). This may be related to the relatively mature early intervention system and social support for children with autism in western countries (36). In contrast, in China, these resources may be relatively lacking, leading to children with autism facing more challenges in their eating behaviors (37). Additionally, the high dietary behavioral issues reported in BAMBI in the ASD group (76.7%) may be due to response bias, as parents of those children with dietary behavioral issues tend to respond to the questionnaire survey compared to parents of children with no such issues, which deserves further investigation.

Finally, linear regression model suggested that BAMBI scores have a statistically significant predictive effect on GI symptoms in ASD cases. The dietary behavior of children with autism may be a target for comprehensive intervention (38) and is an important aspect of behavioral therapy (39). Due to the dietary behaviors of individuals with ASD often occurring at home, training in Applied Behavior Analysis (ABA) and Reflective Functioning (RF) for parents of children with ASD is more important (40).

Several limitations warrant further discussion. Firstly, although our data suggests that the validity and reliability of BAMBI are acceptable, the third subscale was not included in the subsequent analysis, and a larger sample size may be needed in the future to further validate and optimize the BAMBI scale. Secondly, dietary behavior habits are related to dietary habits, but we didn't collect family food logs to conduct further analysis on the dietary differences. Thirdly, due to the small sample size, a larger study is needed to verify some of the observations.

In summary, BAMBI is a validated tool for assessing dietary behaviors in autistic children in China. Compared with their typically developing peers, ASD children presented more dietary behavior problems. This study emphasizes the identification of abnormal eating behaviors in early childhood for early intervention.

## Data Availability

The raw data supporting the conclusions of this article will be made available by the authors, without undue reservation.
